# Mitochondrial Caseinolytic Protease P: A Possible Novel Prognostic Marker and Therapeutic Target in Cancer

**DOI:** 10.3390/ijms22126228

**Published:** 2021-06-09

**Authors:** Antonella Cormio, Francesca Sanguedolce, Vito Pesce, Clara Musicco

**Affiliations:** 1 Department of Biosciences, Biotechnologies and Biopharmaceutics, University of Bari, Via Orabona, 4, 70125 Bari, Italy; antonella.cormio@uniba.it (A.C.); vito.pesce@uniba.it (V.P.); 2 Department of Clinical and Experimental Medicine, University of Foggia, Viale Pinto, 1, 71122 Foggia, Italy; 3 CNR, Institute of Biomembranes, Bioenergetics and Molecular Biotechnologies (IBIOM), Via Amendola 122/O, 70126 Bari, Italy; c.musicco@ibiom.cnr.it

**Keywords:** mitochondrial protease ClpP, mitochondrial quality control, ClpP activators, ClpP inhibitors, OXPHOS

## Abstract

Caseinolytic protease P (ClpP) is a mitochondrial serine protease. In mammalian cells, the heterodimerization of ClpP and its AAA+ ClpX chaperone results in a complex called ClpXP, which has a relevant role in protein homeostasis and in maintaining mitochondrial functionality through the degradation of mitochondrial misfolded or damaged proteins. Recent studies demonstrate that ClpP is upregulated in primary and metastatic human tumors, supports tumor cell proliferation, and its overexpression desensitizes cells to cisplatin. Interestingly, small modulators of ClpP activity, both activators and inhibitors, are able to impair oxidative phosphorylation in cancer cells and to induce apoptosis. This review provides an overview of the role of ClpP in regulating mitochondrial functionality, in supporting tumor cell proliferation and cisplatin resistance; finally, we discuss whether this protease could represent a new prognostic marker and therapeutic target for the treatment of cancer.

## 1. Introduction

In eukaryotic cells, mitochondria are crucial organelles since they produce ATP for several cellular activities through the oxidative phosphorylation process. Therefore, mitochondria are the fulcrum of metabolic pathways and act as regulators of apoptosis, signal transduction and intracellular calcium levels [1,2]. Mitochondria contain small DNA molecules (mtDNA) of around 16569 bp, which code for thirteen subunits of respiratory chain complexes, two rRNAs (12S and 16S) and twenty-two tRNAs [3].

Since mitochondrial respiratory chain is the main source of reactive oxygen species (ROS), it was hypothesized that defective mitochondrial respiratory complexes, especially complex I, might induce increased ROS production [4], resulting in accumulation of oxidative damages to mtDNA, lipids and proteins [5,6,7,8] leading to mitochondrial dysfunctions. In this setting, the mechanisms of mitochondrial quality control intervene by removing oxidative damages; among them, mitochondrial proteases have a central role in maintaining protein homeostasis, namely the Lon protease, Caseinolytic protease P (ClpP), the i-AAA, and m-AAA proteases; the first two are present in the mitochondrial matrix, and the latter in the inner mitochondrial membrane [9,10,11]. These proteases degrade proteins located in all mitochondrial compartments, including subunits of respiratory complexes and translocases.

ClpP is an evolutionarily conserved serine protease, present in prokaryotes and in the mitochondria and chloroplasts. The gene encoding for ClpP is located on chromosome 19, and the protein consists of 277 amino acid residues of the serine protease domain and 56-residues of the mitochondrial N-terminal targeting sequence. In mammals, it forms a heterodimeric complex called ClpXP with its AAA+ ClpX chaperone. This complex consists of two heptameric rings of ClpP subunits that form a central barrel capped at each end by hexameric rings of ClpX subunits. Each ClpP subunit has an active internal catalytic cleavage site (Ser153, His178, and Asp227 residues) [12,13].

The proteolysis occurs in two steps: ClpX recognizes specific motifs in the substrates, binds and partially unfolds the target proteins in an ATP-dependent process, then delivers the proteins to the ClpP proteolytic chamber for degradation. ClpX exerts ATPase activity in the mammalian ClpXP complex [14,15].

ClpPX complex plays regulatory roles within multiple biological pathways, namely “mitochondrial unfolded protein response (UPRMT)” [16], heme biosynthesis by degrading the mitochondrial 5-aminolevulinate synthase ALAS1 and the erythroid-specific ALAS2 [17], PINK1 degradation [18], control of mitochondrial fusion and fission (mitochondrial dynamics) and mitochondrial autophagy (mitophagy) [19], maintenance of the mtDNA by the stabilization of the mitochondrial transcription factor A (TFAM) [20]. Furthermore, ClpP seems to be a crucial regulator of ribosomal assembly and mitochondria translation. It degrades ERAL1, a putative 12S rRNA chaperone, therefore the lack of ClpP induces an increase of ERAL1 that binds to ribosomal 28S particles, preventing the assembly of 55S ribosomes, and ultimately causes mitochondrial translation defects [21].

The aim of this review is to highlight the role of ClpP in regulating mitochondrial quality control, in promoting cell proliferation and cisplatin resistance, and as a new prognostic marker and therapeutic target in human cancer.

## 2. The Mitochondrial Quality Control System

To ensure maximal mitochondrial function, mitochondria carry out different mechanisms for quality control. At the organelle level, damage activates mitochondrial biogenesis (de novo synthesis of mitochondria), mitochondrial dynamics and mitophagy [22,23,24,25]. To preserve mitochondria from ROS-damage at DNA and protein level, antioxidant enzymes, DNA repair mechanisms, protein folding, proteases and UPRMT are active [26,27].

The UPRMT was initially reported in mammalian cells expressing a misfolded mitochondrial protein but has been better characterized in C. elegans [28]. UPRMT involves the increased transcription of proteases, mitochondrial chaperones and antioxidant enzymes; it is induced by alteration of mitochondrial dynamics and respiratory chain, accumulation of misfolded proteins, mtDNA mutations, increase of ROS, inhibition of mitochondrial chaperones and proteases [29,30].

In the ATF5-UPRMT axis, ClpXP complex degrades damaged (unfolded- misfolded) proteins and these short peptides are transported out of the mitochondria into the cytoplasm activating the Transcription Factor Associated with Stress 1 (ATFS-1/ATF5). This factor interacts with the Homeobox domain-containing protein (DVE-1) and ubiquitin-like 5 (UBL-5) in the nucleus, stabilizing chromatin and activating the transcription of genes involved in mitochondrial proteostasis [16,31].

Summing up, ClpP actively participates in protein homeostasis and in maintaining mitochondrial quality control, however, the mechanisms of action of this protease in mammals are not completely clear. Studies on this topic should be addressed.

## 3. The Role of Increased ClpP Expression as a Prognostic Marker in Human Cancer

Many studies demonstrated an increased expression of ClpP in different human cancers.

Cole et al. [32] observed an increased ClpP expression in 45% of the primary acute myeloid leukemia (AML) samples compared to normal hematopoietic cells from healthy individuals. However, the authors reported that high ClpP levels were not associated to CD34 expression, remission attainment, overall or event-free survival, or remission duration, morphologic subtype, cytogenetic risk group, or mutational profile. The analysis of the Cancer Cell Line Encyclopedia database (https://portals.broadinstitute.org/ccle, accessed on 15 May 2021) revealed that ClpP was overexpressed in AML cells, as well as in other hematologic cancers like chronic myeloid leukemia, multiple myeloma and lymphomas. The authors suggested that ClpP is upregulated in AML mitochondria as a consequence of stress. Indeed, in primary AML samples, higher levels of ClpP correlated to higher expression level of UPRMT-related genes. Moreover, leukemic cells showed a great dependency on oxidative phosphorylation (OXPHOS) and an increased mitochondrial mass [33].

Seo et al. [34] demonstrated the overexpression of ClpP in several solid tumors (bladder, prostate, uterus, liver, colon, thyroid, lung, breast, ovary, testis, stomach, lymph nodes and central nervous system) by immunohistochemical analysis. In addition, ClpP showed higher levels of expression in metastatic non-small-cell lung cancer, compared to non-metastatic lesions, and especially in brain metastases. Bioinformatic analysis of the PrognoScan database (website: http://www.prognoscan.org, accessed on 15 May 2021) revealed that ClpP expression correlated with negative patient outcome in about 60% of analyzed datasets; high levels of ClpP expression were associated with shorter distant metastasis-free survival and relapse-free survival in patients with breast carcinoma and uveal melanoma, and in patients with lung adenocarcinoma, respectively [34].

An increase in ClpP expression level has been described in type I endometrial cancer (EC) tissues as compared to normal controls [35]. Interestingly, this increase was more evident in EC tissue harboring pathogenic mtDNA mutations and complex I deficiency. It can be envisioned that this increase may be a defense mechanism of cancer cells to complex I deficit and to increased oxidative stress, which have been both observed in EC [36,37]. In this oxidative stress condition, the increased ClpP expression may contribute to removing damaged mitochondrial proteins and maintaining mitochondrial function. Accordingly, in bladder cancer, a tumor characterized by oxidative stress condition, we observed higher ClpP expression levels compared to corresponding adjacent normal tissues (Cormio et al., unpublished results).

Recently, ClpP expression was measured in human breast cancer (BC) tissues and corresponding adjacent normal tissues, as well as in seven human BC cell lines and two normal mammary epithelial cell lines. The authors reported an upregulation of ClpP in both tumor cells and tissues. Silencing of ClpP in BC cell lines considerably reduced cell growth, migration and invasion, and induced cell death, thus indicating a possible crucial role of ClpP in BC tumorigenesis. Moreover, the analysis of the Cancer Genome Atlas (TCGA) and Kaplan–Meier-plotter database revealed a significant correlation between the expression level of ClpP and clinical-pathological characteristics of BC patients (T stage, estrogen receptor expression, and poorer recurrence-free survival (RFS)) [38].

Overall, these findings indicate that ClpP overexpression seems to be a common feature of different cancers (Table 1) and, in some solid tumors, may be associated with a more aggressive disease, namely presence of metastases and poor RFS. Unfortunately, the prognostic role of ClpP was not widely studied in different solid and hematologic cancers, there are grounds to state that this topic should be better explored to evaluate whether this protease could be considered a novel prognostic marker.

## 4. The Influence of Loss of ClpP on Mitochondrial Respiratory Complexes and Cell Viability

Several ClpP targets have been recognized, including subunits of the respiratory and pyruvate dehydrogenase complexes as well as of the tricarboxylic acid cycle enzymes, suggesting a central role of ClpP in energetic metabolism [39].

Lower rates of mitochondrial respiration, oxygen consumption, membrane potential, as well as increased generation of ROS and impaired myoblast differentiation and cell proliferation, have all been reported in deficient ClpP muscle cells [40]. Moreover, these cells presented high phosphorylation level of initiation factor 2 alpha, leading to inhibition of translation initiation, and a reduced response to UPRMT induction.

In dopaminergic SH-SY5Y neuronal cells the downregulation of ClpP by RNA interference induced an increase in mitochondrial misfolded and unfolded proteins, suppressed mitochondrial respiratory activity, increased mitochondrial oxidative damage and caused cell death. Overexpression of ClpP increased the level of superoxide dismutase-2 in these cells reducing mitochondrial oxidative stress [41].

Cole et al. [32] demonstrated that the knockdown of ClpP in AML cells induced accumulation of malfunctioning succinate dehydrogenase (SDHA) subunit of respiratory complex II, reduced the enzymatic activity of this complex and increased mitochondrial ROS production. Thus, these results suggest that SDHA is a target of ClpP, and that ClpP has a key role in preserving complex II activity in AML cells. Moreover, knockdown of ClpP in mtDNA depleted osteosarcoma cells (143B Rho 0), cells not depending on OXPHOS, had little effect on cell viability compared to osteosarcoma 143B cell with normal mtDNA content. This argues that ClpP regulates mitochondrial function especially in cells depending on OXPHOS.

A proteomic study in mitochondria of prostate adenocarcinoma PC3 cells showed that survivin co-immunoprecipitated with ClpP, TRAP-1 and SDHB subunit of respiratory complex II. ClpP silencing in these cells induces an increase of misfolded subunits of mitochondrial respiratory complex II but not of complexes I, III, IV or V. The knockdown of ClpX or ClpP in prostate cancer cell significantly reduced complex II activity and overall ATP production and increase glucose consumption and lactate production, suggesting a compensatory increase in glycolysis. Moreover, the silencing of ClpP causes increased oxidative damages and the induction of autophagy and apoptosis [34].

Recently, it was demonstrated that ClpXP complex can identify altered core N-module proteins of respiratory complex I (CI) and then disassemble and degrade them, allowing for an exchange with new N-modules subunits in the pre-existing CI [42]. N-module is the primary site of CI ROS production, making it particularly prone to oxidative damage [43]. The replacement of the N-module by the mitochondrial protein quality control system is needed to ensure CI function. Therefore, when ClpP is not expressed in heart mitochondria, N-modules cannot be replaced by ClpXP complex, and the accumulation of inactive CI subunits impairs electron flow.

Gispert et al. [44] observed a minor respiratory dysfunction only in muscle and liver mitochondria of ClpP null mice. These mice have a partially embryonic lethality and surviving pups exhibit growth retardation, female and male infertility and die prematurely. Accordingly, Cole et al. [32] created constitutive ClpP-deficient mice that were viable with normal hematopoiesis but showed infertility and hearing loss.

A study of Szczepanowska et al. [21] on ClpP−/− mouse showed a reduction of respiratory complex IV subunits in heart, and of subunits of complex III and IV in skeletal muscles, but no changes in liver. The authors hypothesized that loss of ClpP leads to a deficit of OXPHOS due to altered mitochondrial protein synthesis, owing to reduced level of assembled mitochondrial ribosomes. This effect is stronger in tissues with higher mitochondrial activity.

All these data indicate that the genetic modulation of ClpP in different cell types may affect mitochondrial respiratory complexes activities, mitochondrial metabolism, and finally cell viability. However, ClpP−/− mice are viable and showed only infertility and a slight decrease in size. Likewise, human patients harboring mutations in ClpP acquired only hearing loss and infertility [45], thus suggesting that the loss of ClpP is not incompatible with life but seems to affect mostly cells relying on oxidative phosphorylation.

## 5. The Chemical Modulation of ClpP: A Promising Anticancer Therapy

As previously reported, many cancers showed increased expression levels of ClpP, and the silencing of this protease significantly impairs mitochondrial OXPHOS and inhibits cell proliferation. Considering these results, recent studies try to identify and characterize new molecules that can target and modulate ClpP activity to selectively kill cancer cells.

Earlier studies on ClpP inhibitors were developed in bacteria, since it was observed that inhibition of ClpP activity also reduces their infection rate. The first ClpP inhibitors discovered in bacteria were the trans-ß-lactones. These molecules inhibit ClpP activity through covalent modification of Ser 153 residue [46].

Moreover, it was reported [32] that a synthetic ß-lactone, A2-32-01, destroyed mostly AML cell lines and primary AML samples with high ClpP expression compared to non-malignant hematopoietic cells and AML cells with low ClpP expression. Therefore, a positive correlation between ClpP expression and sensitivity to A2-32-01 was found in AML cells. The authors suggested that the inhibition of ClpP by A2-32-01 leads to the accumulation of damaged and misfolded respiratory chain proteins, resulting in impaired oxidative phosphorylation and ultimately cell death. A2-32-01 also decreased the proliferation of wild-type osteosarcoma 143B cells, but not of Rho 0 counterparts. A2-32-01 reduced cell proliferation in mice xenografted with OCI-AML2 cells, without inducing a toxic effect on liver, muscle and kidney. Furthermore, tumor masses resected from mice treated with A2-32-01 showed low respiratory chain complex II activity and low ClpP activity compared to controls. These data confirm the possible use of ClpP inhibitors for the treatment of AML. Unfortunately, A2-32-01 is not a stabile compound, therefore it is unsuitable for clinical development.

Phenyl ester compound AV167 and modified analogues TG42, TG43 inhibit human ClpP proteolytic activity [47], and induce apoptosis and decreased cell migration of hepatocyte-derived carcinoma cells [48]. Boron-based peptidomimetics, such as α-aminoboronic acids were also identified as ClpP inhibitors [49]. However, no study on cell culture is available yet.

Different compounds that activate ClpP have been also discovered. In contrast to ClpP inhibitors, ClpP activators replace ClpX, open the pore of the ClpP protease and then improve proteolytic activity without the presence of ClpX. Interestingly, also ClpP activation induced tumor cell death by inducing an uncontrolled proteolysis of ClpP substrates, as well as respiratory chain proteins, with subsequent impairment of OXPHOS and apoptosis.

Wong et al. [14] demonstrated that Acyldepsipeptides (ADEP-41) may activate the ClpP protease. In Embryonic kidney cells (HEK293 T-Rex), ADEP induced mitochondrial fragmentation and apoptosis and abolished OXPHOS function. Conversely, in the same cell type with ClpP knock out, ADEP had no effect on cell growth. Moreover, ADEP-treated wild-type cells induce an increase in basal acidification rate suggesting that these cells have lost OXPHOS and rely on glycolysis for energy metabolism. ADEP induced cell death in cervical carcinoma cell (HeLa), HeLa T-Rex Cervical carcinoma, osteosarcoma cells (U2OS) and undifferentiated neuroblastoma cell (SH-SY5Y) as well. It has been shown that ADEP sensitivity differs barely among the analyzed cell lines.

The first-in-class imipridone molecule, ONC201, has anti-proliferative and pro-apoptotic effects in numerous solid tumors and hematological malignancies [50,51]. Ishizawa et al. [52] identified ONC201 as a ClpX-independent activator of human ClpP. It docks directly into the hydrophobic pocket of ClpP forming a barrel shaped oligomer and opening the proteolytic chamber from 12 A to 17 A. ClpP activation causes the degradation of mitochondrial proteins including subunits of respiratory complexes leading to decreased activity of complexes I, II and IV and basal oxygen consumption rate, and to increased ROS production in wild-type, but not in ClpP mutated Z138 cells. ONC201 and the more potent analogue ONC212 induced lethality in leukemia and lymphoma cell lines as well as in primary AML cells, but it does not hit non-tumor cells.

Greer et al. [53] showed in breast cancer cells a positive correlation between the antiproliferative activity of ONC201, reduced oxidative phosphorylation and the number of functional mitochondria. Indeed, ONC201 has been shown to decrease mtDNA, and suppress the expression of many nuclear and mtDNA-encoded genes. Interestingly, ONC201 was ineffective in cells not dependent on mitochondrial respiration, such as Rho 0 cells.

Graves et al. [54] tried out different new ONC201 analogues (TR compounds) for cell viability on Human Triple Negative Breast Cancer cell lines, SUM159 and MDA-MB-231. These compounds, binding to ClpP, were more strong activators of ClpP than ONC201, and, like ONC201, inhibited cell proliferation and induced the integrated stress response protein ATF4. SiRNA knockdown of ClpP in SUM159 cells decreased the response to ONC201 and TR compounds. The mechanisms by which these compounds induce cell death is not entirely clear, however, it looks like that they induce alterations in mitochondrial morphology, loss of TFAM, inhibition of oxidative phosphorylation and lactic acid formation.

Pruus at al. [55] demonstrated that ONC201 significantly decreased mitochondrial respiration and complex I and complex II expression and upregulated the glycolytic rate in U251 and A172 glioblastoma cells. Moreover, when imipridone was combined with 2-Deoxyglucose, an inhibitor of glycolysis, the low ATP production induced a reduction of tumor cell growth and migration. These molecules pass through the blood–brain barrier and have been used in clinical trials with no adverse effects. However, the authors did not test the effects of these agent on ClpP activity. Therefore, CNS tumors are particularly sensible to imipridones due to their relative dependencies on mitochondrial OXPHOS; moreover, ClpP has been reported as the most significant predictive biomarker of response to treatment to ONC201 according to a recent meta-analysis [56].

**Figure 1:**
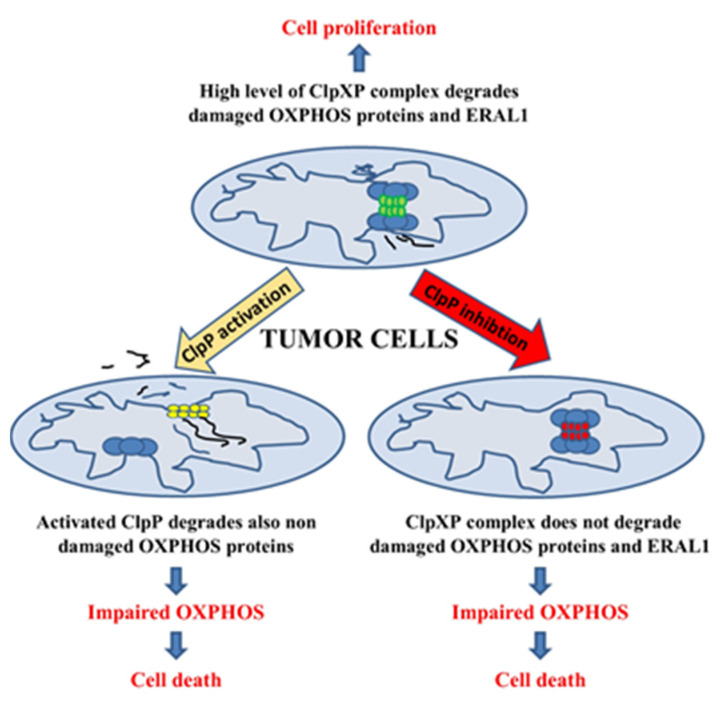
Effects of chemical modulation of ClpP on tumor cells relying on OXPHOS. High levels of ClpPX complex allow the removal of damaged OXPHOS proteins and ERAL1. This ensures the integrity of the respiratory chain and mitochondrial ribosome assembly, thus maintaining mitochondrial functionality and promoting tumor cell proliferation. ClpP activation causes the degradation of non-damaged OXPHOS proteins leading to impaired OXPHOS, mitochondrial dysfunction, and ultimately cell death; ClpP inhibition does not allow the removal of damaged OXPHOS proteins and ERAL1 leading to impaired OXPHOS, altered ribosome assembly, mitochondrial dysfunction, and ultimately cell death.

All in all, we can argue that either the activation or the inhibition of ClpP would offer novel mechanisms to selectively kill tumor cells relying on OXPHOS, as reported in Figure 1.

Several important questions should be addressed. Although some of these molecules are well tolerated in patients, we do not know yet which dose can effectively modulate ClpP activity and destroy tumor cells. Furthermore, it would be interesting to investigate the basal expression of ClpP or the presence of ClpP mutations in tumor cells, since the real amount of active ClpP may affect the sensibility to these molecules.

## 6. Mitochondria and Cisplatin Resistance: The Involvement of ClpP

Cisplatin is one of the most effective and commonly chemotherapy drugs used to treat many solid tumors. This drug interacts with nuclear DNA, mtDNA and proteins [57]. Cisplatin caused a decrease in mitochondrial biogenesis, mass and membrane potential, leading to a reduction in ATP content, and an increase in ROS production and consequently oxidative damages [58].

Recently, several studies have highlighted that mitochondria are both the target of cisplatin and the pivot of cisplatin resistance since they try to evade death pathway in stress condition [59]. In particular, mitochondrial fusion seems to stimulate cell survival to cisplatin, because of an improved mitochondrial function, through an efficient production and transport of ATP [60]. Conversely, a more fragmented mitochondrial network makes cancer cells more sensitive to cisplatin [61] by promoting apoptosis. Moreover, the elimination of damaged mitochondria seems to be a pro-survival mechanism, in that cisplatin-resistant ovarian cancer cells showed increased mitophagy and when these cells were treated with several autophagy inhibitors showed increased sensitivity to cisplatin [62].

Interestingly, ClpP overexpression desensitizes cells to cisplatin, conversely, its silencing makes tumor cells more sensitive to cisplatin. Indeed, ClpP depletion induces the increase in cisplatin-DNA adducts, but the overexpression of an inactive form of ClpP had no effect. The overexpression of ClpP increased the levels of ATP7A and ATP7B, copper transporting pumps, that are involved in the removal of cisplatin from cells, thus suggesting a possible role of this protease also in cisplatin cellular accumulation [63].

One can speculate that ClpP, together with mitophagy, may help cells to maintain mitochondrial functionality by repairing or resisting to the damages caused by cisplatin, therefore, targeting ClpP could represent a new mechanism to make cancer cells sensitive to cisplatin, and the expression level of ClpP in tumor tissues could represent a potential predictive biomarker in this setting. However, additional in vitro and in vivo studies are necessary to clarify this point.

## 7. Conclusions

Reprogramming of energy metabolism in tumor cells has been recognized over the past sixty years. The most described metabolic phenotype in cancer cells is the Warburg effect. Cancer cells exhibit high glucose metabolism producing an excess of lactate even in aerobic conditions [64]. During cell proliferation, the increase in glycolytic rate plays a pivotal role in sustaining the large-scale anabolic processes. However, some reports showed that cancer cells also increase dependence on oxidative phosphorylation [65,66], suggesting that targeting mitochondrial respiratory chain could selectively destroy tumor cells.

ClpP, maintaining the integrity of the oxidative phosphorylation system, could represent a new potential therapeutic target for the treatment of cancer. Indeed, its inhibition and activation impair OXPHOS and mitochondrial functionality leading to anti-proliferative effects in cancer cells relying on OXPHOS. However, further studies are needed to better recognize the in vivo effects and toxicity of ClpP inhibitors and activators.

Furthermore, the relationship between ClpP expression and tumor progression, survival, and drugs sensitivity in oncologic patients should be deeply investigated to evaluate whether this protease could represent not only a therapeutic target but also a new prognostic and predictive marker in human cancer.

## Figures and Tables

**Table 1:** Overexpression of ClpP in different cancers.

Tumor Type	Sample	Ref.
Acute Myeloid Leukemia	non-solid tumor	[32]
Uterus	solid tumor	[34,35]
Bladder	solid tumor	[34], Cormio et al., unpublished
Prostate	solid tumor	[34]
Lung	solid tumor	[34]
Liver	solid tumor	[34]
Colon	solid tumor	[34]
Thyroid	solid tumor	[34]
Ovary	solid tumor	[34]
Testis	solid tumor	[34]
Stomach	solid tumor	[34]
Lymph nodes	solid tumor	[34]
Central nervous system	solid tumor	[34]
Uveal melanoma	solid tumor	[34]
Breast	solid tumor, ll lines (MCF-7, ZR-75-1, MB-231)	[34,38]

## Data Availability

Not applicable.
